# Disability Acceptance and Unmet Healthcare Needs Among Persons with Disabilities in Korea: A Seven-Wave Random-Intercept Cross-Lagged Panel Study

**DOI:** 10.3390/healthcare14152317

**Published:** 2026-07-31

**Authors:** Joungmin Kim, Sanggyeong Lee, Yaena Ha

**Affiliations:** 1 Healthcare Service School, Soonchunhyang University, 22 Soonchunhyang-ro, Asan-si 31538, Republic of Korea; 2 Department of ICT Convergence, Graduate School, Soonchunhyang University, 22 Soonchunhyang-ro, Asan-si 31538, Republic of Korea; 3 Emotional and Intelligent Child Care System Convergence Research Center, Soonchunhyang University, 22 Soonchunhyang-ro, Asan-si 31538, Republic of Korea

**Keywords:** disability acceptance, unmet healthcare needs, longitudinal study, random intercept cross-lagged panel model, Disability and Life Dynamics Panel, Korea, psychosocial adaptation, healthcare access

## Abstract

**Highlights:**

**What are the main findings?**

- This study is the first to apply a Random Intercept Cross-Lagged Panel Model (RI-CLPM) over seven years, revealing a significant negative trait-level correlation (*r* = −0.132) between disability acceptance and unmet healthcare needs among newly registered persons with disabilities in South Korea.
- At the within-person level, reciprocal prospective associations emerged selectively rather than continuously, with significant associations observed during transitions overlapping with the COVID-19 pandemic period and its immediate aftermath; all significant associations survived false discovery rate correction.

**What are the implications of the main findings?**

- The findings suggest that healthcare access interventions for populations with disabilities should integrate psychosocial adaptation support, such as counseling and peer programs, alongside traditional efforts to remove structural and financial barriers.
- The temporal clustering of cross-lagged associations underscores the critical need for disability-responsive healthcare contingency planning and continuity-of-care protocols during periods of macro-level systemic stress or public health emergencies.

**Abstract:**

**Background:** Persons with disabilities experience persistent unmet healthcare needs, but the longitudinal interplay between psychological adaptation to disability and healthcare access has rarely been examined using analytic approaches that distinguish stable between-person differences from within-person changes over time. This study investigated the bidirectional longitudinal associations between disability acceptance and unmet healthcare needs over seven years. **Methods:** We analyzed seven annual waves (2018–2024) of the Korean Disability and Life Dynamics Panel (DLDP), a nationally representative cohort of 6121 newly registered persons with disabilities. Disability acceptance was measured using the 12-item Disability Acceptance Scale (Cronbach’s α range: 0.808–0.823), whereas unmet healthcare needs were assessed using a single dichotomous item at each wave. A Random Intercept Cross-Lagged Panel Model (RI-CLPM) was estimated using maximum. **Results:** The freely estimated RI-CLPM demonstrated good model fit: Comparative Fit Index (CFI) = 0.996; Tucker–Lewis Index (TLI) = 0.994; Root Mean Square Error of Approximation (RMSEA) = 0.026, 90% confidence interval (CI) [0.023, 0.029]; and Standardized Root Mean Square Residual (SRMR) = 0.020. At the trait level, the random intercept correlation between disability acceptance and unmet healthcare needs was statistically significant and negative (*r* = −0.132, *p* = 0.001). At the within-person level, cross-lagged associations were small but consistently negative; after false discovery rate correction, four of the twelve cross-lagged paths remained statistically significant. The path from disability acceptance to subsequent unmet healthcare needs was significant at two transitions (Wave 3 to Wave 4: *β* = −0.057, *p* = 0.002; Wave 5 to Wave 6: *β* = −0.065, *p* = 0.008), whereas the reverse path was significant at two transitions (Wave 2 to Wave 3: *β* = −0.013, *p* = 0.008; Wave 6 to Wave 7: *β* = −0.050, *p* = 0.008). These transitions coincided with the COVID-19 pandemic and its aftermath, although formal tests comparing pandemic and non-pandemic transitions were not statistically significant. **Conclusions:** Disability acceptance and unmet healthcare needs were inversely associated at the trait level among Korean persons with disabilities, and reciprocal within-person associations emerged selectively during periods of healthcare system disruption. Healthcare access interventions for persons with disabilities may benefit from integrating psychosocial adaptation support alongside efforts to reduce structural barriers to care.

## 1. Introduction

Persons with disabilities experience disproportionately high rates of unmet healthcare needs across high-income countries, including South Korea [1,2,3]. Unmet healthcare needs, defined as instances in which an individual is unable to receive adequate medical care when needed, reflect a critical failure of healthcare systems to ensure equitable access for vulnerable populations. The persistence of unmet healthcare needs among persons with disabilities is well documented in cross-sectional surveys and short-term longitudinal studies, with reported prevalence ranging from 4% to 12% depending on disability type, severity, and national context [2,3]. Although structural barriers such as financial constraints, physical accessibility, transportation, and provider attitudes have been extensively examined, less attention has been paid to the role of psychological adaptation to disability as a longitudinal correlate of healthcare access disparities.

Disability acceptance, the psychosocial process through which individuals come to value their lives in the presence of disability rather than in opposition to it, has been theorized for over five decades as a central construct in adaptation to acquired disability [4,5,6]. The Acceptance of Disability Scale, originally developed by Linkowski [4] and later refined by Groomes and Linkowski [5], operationalizes four conceptual domains: enlargement of values, subordination of physique, containment of disability effects, and transformation of comparative-status to asset values. Higher levels of disability acceptance have been linked to better mental health [6], greater quality of life, more proactive engagement with rehabilitation services, and stronger self-advocacy behavior. These positive associations suggest that disability acceptance may also be linked to healthcare access through the propensity to seek timely medical care, to communicate effectively with healthcare providers, and to persist in the face of access barriers.

Conversely, theoretical accounts of psychosocial adaptation under chronic stress suggest that experiences of unmet healthcare needs may erode disability acceptance over time. When individuals are unable to obtain needed medical care, the resulting frustration, perceived loss of control, and reinforcement of negative disability-related schemas may undermine the cognitive and emotional processes that support adaptive disability acceptance. This reciprocal possibility implies that disability acceptance and unmet healthcare needs may be linked through a bidirectional rather than unidirectional process, with implications for both intervention targeting and theoretical understanding.

Despite the theoretical plausibility of bidirectional dynamics, the empirical literature on the longitudinal interplay between disability acceptance and healthcare access remains sparse. Prior research using the Korean Disability and Life Dynamics Panel (DLDP), a nationally representative cohort of newly registered persons with disabilities, has documented persistent unmet healthcare needs and identified functional limitations as predictors of these needs [2,7], but has not examined disability acceptance as either an antecedent or consequence of unmet care. Moreover, existing DLDP-based studies have generally relied on traditional cross-lagged panel models, generalized estimating equations, or simple trend analyses, none of which separate stable between-person differences from within-person dynamics. As Hamaker, Kuiper, and Grasman [8] demonstrated, conventional cross-lagged panel models conflate these two sources of variation, so that estimated cross-lagged paths reflect a mixture of within-person change and between-person trait differences. This methodological limitation is particularly consequential when both constructs of interest (here, disability acceptance and unmet healthcare needs) are themselves shaped by enduring inter-individual differences.

The Random Intercept Cross-Lagged Panel Model (RI-CLPM) developed by Hamaker and colleagues [8] and extended by Mulder and Hamaker [9] addresses this limitation by explicitly partitioning observed variance into a stable between-person component (modeled as latent random intercepts) and a within-person component (modeled as time-specific latent deviations from each individual’s expected trajectory). Cross-lagged paths in the RI-CLPM are therefore interpretable as within-person prospective effects, the extent to which an individual’s deviation from their own typical level on one construct is associated with subsequent deviations on the other after controlling for stable trait differences. This specification is widely regarded as an appropriate framework for examining reciprocal longitudinal associations with panel data.

The present study applies the RI-CLPM to seven annual waves (2018–2024) of the Korean DLDP to examine the bidirectional longitudinal associations between disability acceptance and unmet healthcare needs. The seven-year observation window encompasses the pre-pandemic period (Waves 1–2), the COVID-19 pandemic period (Waves 3–5), and the post-pandemic recovery period (Waves 6–7), enabling examination of whether reciprocal dynamics shift across periods of differential healthcare system stress. Three research questions guide the analysis: (a) Are disability acceptance and unmet healthcare needs associated at the stable trait level, after partialling out within-person fluctuations? (b) Is within-person variation in disability acceptance prospectively associated with subsequent within-person variation in unmet healthcare needs? (c) Is within-person variation in unmet healthcare needs prospectively associated with subsequent within-person variation in disability acceptance? Based on the theoretical considerations outlined above, we hypothesized that the trait-level association would be negative (Hypothesis 1) and that both cross-lagged paths would be negative (Hypotheses 2 and 3), reflecting reciprocal protective dynamics.

## 2. Materials and Methods

### 2.1. Study Design and Data Source

This study analyzed seven annual waves of secondary data from the DLDP, a nationally representative longitudinal survey administered by the Korea Disabled People’s Development Institute (KODDI). The DLDP follows a cohort of 6121 newly registered persons with disabilities who completed their disability registration under the Korean Act on Welfare of Persons with Disabilities between 1 January 2015 and 31 December 2017, with annual face-to-face follow-up assessments beginning in 2018. The panel employs a stratified two-stage sampling design using disability type, disability severity, and sex as stratification variables, thereby ensuring national representativeness of newly registered persons with disabilities in South Korea. The inclusion criteria were individuals newly registered within this period who consented to participate in the longitudinal panel survey; individuals residing in institutional care settings were excluded. Data were collected through annual face-to-face interviews conducted by trained professional interviewers using Tablet-Assisted Personal Interviewing (TAPI). The DLDP is approved as Korean government statistics by Statistics Korea (Approval No. 438001), and the data are released for academic research purposes following KODDI’s annual data dissemination conference.

For the present analysis, we used Wave 1 (2018) through Wave 7 (2024), covering a seven-year observation period. Response retention across the seven waves was 100% at Wave 1 (*n* = 6121), 90.3% at Wave 2 (*n* = 5527), 85.9% at Wave 3 (*n* = 5259), 82.1% at Wave 4 (*n* = 5024), 80.1% at Wave 5 (*n* = 4904), 78.4% at Wave 6 (*n* = 4796), and 76.8% at Wave 7 (*n* = 4702). A total of 4283 participants (70.0% of the original cohort) completed all seven waves.

To maximize the use of available information, missing data were handled using full-information maximum likelihood (FIML) estimation, which is widely recommended for longitudinal structural equation modeling because it uses all available observations and provides unbiased parameter estimates under the assumption that data are missing at random (MAR) [10]. To evaluate this assumption, attrition analyses were conducted prior to model estimation (see Section 3.4), which indicated that missingness was related to observed baseline characteristics rather than to the study outcome, a pattern consistent with MAR.

### 2.2. Measures

#### 2.2.1. Disability Acceptance

Disability acceptance was assessed using the 12-item Disability Acceptance Scale included in the DLDP, derived from the broader Acceptance of Disability Scale framework originally developed by Linkowski [4]. It was later refined by Groomes and Linkowski [5]. The Korean adaptation of the 12-item scale used in the DLDP was developed by Kang, Park, and Gu [11]. Respondents indicated their level of agreement with each item on a 4-point Likert scale (1 = “strongly disagree” to 4 = “strongly agree”). Items addressed psychosocial adaptation domains such as self-evaluation, enlargement of values, containment of disability effects, and acceptance of physical limitations. Four items (Items 1, 3, 7, and 12) were negatively worded and reverse-coded before score computation. A mean score across the 12 items was calculated for each wave, with higher scores indicating greater acceptance of disability. Internal consistency was good across all seven waves (Cronbach’s α range: 0.808–0.823).

Although confirmatory factor analysis at Wave 1 suggested a suboptimal fit for a unidimensional model, χ^2^(54) = 5323.58, *p* < 0.001, comparative fit index (CFI) = 0.782, and RMSEA = 0.126, parallel analysis supported a four-factor solution in which two factors comprised primarily reverse-coded items. This pattern suggested wording-related method effects rather than clear substantive multidimensionality [12]. Given the high internal consistency of the full scale and consistency with prior RI-CLPM research using manifest composite scores [9], we used the mean score as a manifest indicator of disability acceptance.

#### 2.2.2. Unmet Healthcare Needs

Unmet healthcare needs were assessed at each wave using a single item asking respondents whether, during the past year, they had experienced a situation in which they were unable to receive adequate medical care at a hospital or clinic (Korean original: “During the past year, have you experienced a situation in which you were unable to receive adequate medical care at a hospital or clinic?”). Response options were dichotomous (1 = “yes”; 2 = “no”), and responses were recorded so that 1 indicated the presence of unmet healthcare needs and 0 indicated the absence of unmet healthcare needs. This measurement approach is consistent with prior studies examining unmet healthcare needs among persons with disabilities in Korea using the same panel dataset [2,7].

#### 2.2.3. Covariates

To characterize the sample and facilitate interpretation of the random intercept (between-person) component of the model, we extracted the following Wave 1 covariates from the DLDP: sex (1 = male, 2 = female), age category (three-category classification based on the sampling design), disability type (a seven-category classification based on the national disability registration system under the Korean Act on Welfare of Persons with Disabilities: physical, brain lesion, visual, hearing/speech, intellectual/autistic, psychiatric, and internal/facial), disability severity (the statutory two-level classification applied at registration: 1 = severe, 2 = mild), and educational attainment.

Disability type and severity in the DLDP are anchored to the original enrollment classification (2015–2017) and are treated as time-invariant across waves. Accordingly, subsequent changes in disability classification or severity reassessment are not reflected in the dataset.

Although unmet healthcare needs were modeled as a dichotomous variable, we used robust maximum likelihood estimation (MLR) rather than weighted least squares with mean and variance adjustment (WLSMV) for several reasons. First, MLR with FIML allows retention of all available longitudinal observations. In contrast, WLSMV in longitudinal SEM commonly relies on pairwise present data handling, potentially reducing the effective sample size across seven waves. Second, exploratory WLSMV estimation produced unstable model solutions in the present RI-CLPM specification, likely reflecting the combination of low-prevalence binary outcomes and highly parameterized longitudinal constraints. Third, prior methodological research has shown that ML-based robust estimators provide acceptable parameter recovery and Type I error control for categorical outcomes in structural equation models when sample size is large and the primary analytic interest concerns standardized longitudinal associations rather than probability estimation [13].

### 2.3. Statistical Analysis

#### 2.3.1. Analytic Approach

To examine the bidirectional longitudinal associations between disability acceptance and unmet healthcare needs, we estimated a RI-CLPM as proposed by Hamaker, Kuiper, and Grasman [8] and further elaborated by Mulder and Hamaker [9]. The RI-CLPM extends the traditional cross-lagged panel model by partitioning observed variance into stable between-person differences (captured by latent random intercepts) and within-person fluctuations around each individual’s expected level over time. Cross-lagged paths in the RI-CLPM therefore represent within-person processes, that is, the extent to which deviations from an individual’s own expected level on one construct at time t are associated with deviations from their expected level on the other construct at time t + 1, after accounting for stable between-person differences. This modeling approach is increasingly recommended for examining reciprocal longitudinal associations because it addresses several limitations of the traditional cross-lagged panel model [8].

**Figure 1:**
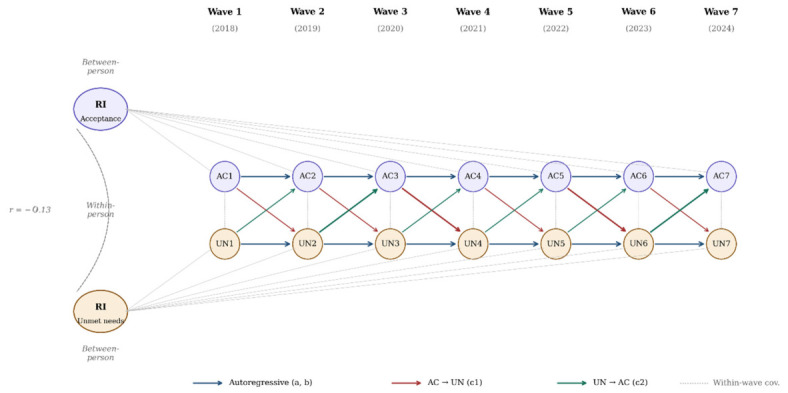
Conceptual Structure of the Freely Estimated Random Intercept Cross-Lagged Panel Model (RI-CLPM). Random intercepts (RI) capture stable between-person differences in disability acceptance and unmet healthcare needs. Within-person latent variables represent wave-specific deviations from each individual’s expected level over time. Blue arrows indicate autoregressive paths; red arrows indicate cross-lagged paths from disability acceptance to subsequent unmet healthcare needs (c1); green arrows indicate reciprocal paths from unmet healthcare needs to subsequent disability acceptance (c2). Gray dashed lines represent within-wave covariances. Thicker arrows denote statistically significant cross-lagged associations.

For each construct (disability acceptance and unmet healthcare needs), we specified a latent random intercept, with all factor loadings constrained to 1.0, to capture the stable, trait-like component. Within-person latent variables were specified at each of the seven waves, each with a single indicator (the observed wave score) and a unit factor loading. Residual variances of the observed indicators were constrained to zero, thereby partitioning variance between the random intercept and within-person latent components. The model included autoregressive paths (from each within-person latent variable to the corresponding variable at the subsequent wave for the same construct), cross-lagged paths (from each within-person latent variable to the subsequent-wave within-person latent variable of the other construct), and within-wave covariances between the two constructs’ within-person variables. The two random intercepts were allowed to covary, whereas covariances between random intercepts and within-person latent variables were constrained to zero, consistent with the RI-CLPM specification [9]. Figure 1 illustrates the conceptual structure of the freely estimated RI-CLPM used in the present study.

#### 2.3.2. Time-Invariant Versus Time-Varying Specification

We initially specified a time-invariant RI-CLPM in which the autoregressive and cross-lagged coefficients were constrained to be equal across all six wave-to-wave transitions, consistent with common practice in longitudinal panel modeling. Although this specification demonstrated acceptable values for the comparative fit index, Tucker–Lewis index (TLI), and root mean square error of approximation, the standardized root mean square residual indicated poor fit (SRMR = 0.229), suggesting residual covariance misfit [14]. A nested likelihood-ratio test comparing this model with a freely estimated specification revealed a significantly better fit for the freely estimated model, with a scaled Δχ^2^(20) = 838.25, *p* < 0.001, and improvements in both CFI (ΔCFI = 0.034) and root mean square error of approximation (ΔRMSEA = −0.046) [14,15]. Accordingly, the freely estimated model was retained as the primary analytic specification, allowing coefficients to vary across the six wave-to-wave transitions.

#### 2.3.3. Estimation and Model Fit

Model parameters were estimated using robust maximum likelihood estimation (estimator = “MLR”), which provides standard errors and test statistics that are relatively robust to non-normality. Because unmet healthcare needs were modeled as a single observed dichotomous variable within the RI-CLPM framework, the MLR estimator was used to obtain robust parameter estimates in a large-sample context. Missing data were handled using FIML.

Model fit was evaluated using multiple indices, with the following criteria indicating acceptable to good fit: CFI ≥ 0.95; TLI ≥ 0.95; RMSEA ≤ 0.06 with the upper bound of the 90% confidence interval (CI) ≤ 0.08; and SRMR ≤ 0.08 [14].

Descriptive and preliminary analyses were conducted using IBM SPSS Statistics version 30 (IBM Corp., Armonk, NY, USA). The RI-CLPM and all supplementary structural equation models were estimated in R version 4.6.0 (R Foundation for Statistical Computing, Vienna, Austria; https://www.r-project.org/, accessed on 20 July 2026) [16] using the lavaan package version 0.6-19 (https://lavaan.ugent.be/, accessed on 20 July 2026) [17], because the robust maximum likelihood, categorical weighted least squares, and full-information maximum likelihood procedures reported in Section 2.3.3 and Section 2.3.4 required this environment. 

#### 2.3.4. Multiple Testing, Sensitivity, and Robustness Analyses

Because twelve cross-lagged paths were freely estimated, we controlled the false discovery rate (FDR) across these tests using the Benjamini–Hochberg procedure [18], with Bonferroni-corrected results reported for comparison. To address the dichotomous nature of the outcome, the cross-lagged structure was re-estimated using a categorical specification (WLSMV estimation with theta parameterization on complete cases, *n* = 4283), and the RI-CLPM was compared with a traditional cross-lagged panel model using AIC and BIC. Targeted longitudinal measurement invariance of the disability acceptance scale was tested across Waves 5 and 6 (configural, metric, and scalar models evaluated against the ΔCFI ≤ 0.01 criterion [15]). Attrition was examined by regressing Wave 7 non-response on baseline unmet healthcare needs, disability acceptance, and sociodemographic covariates, and a covariate-adjusted RI-CLPM including baseline sex, disability severity, age group, and educational attainment as predictors of the random intercepts was estimated. Because the DLDP employs a stratified two-stage sampling design, design-weighted prevalence estimates of unmet healthcare needs were computed for each wave using wave-specific cross-sectional weights; the primary RI-CLPM was estimated without sampling weights, consistent with model-based longitudinal SEM practice. Finally, the robustness of pandemic-related interpretations was evaluated using Wald tests comparing average cross-lagged coefficients between the pandemic era (2019–2022) and non-pandemic transitions.

### 2.4. Ethical Considerations

The DLDP is conducted by the KODDI under the approval of Statistics Korea (Approval No. 438001) and is governed by the Personal Information Protection Act of the Republic of Korea. Written informed consent was obtained from all participants at the time of panel enrollment. The present study analyzed the public-use version of the DLDP, in which personally identifiable information had been removed, and key stratification variables had been de-identified to minimize re-identification risk. Because this study involved secondary analysis of publicly available de-identified data, additional institutional review board approval was not required. The authors acknowledge KODDI as the source institution for the DLDP data.

## 3. Results

### 3.1. Sample Characteristics

The baseline (Wave 1, 2018) sample comprised 6121 newly registered persons with disabilities in South Korea. The sample was predominantly male (55.4%), and more than half of the participants were aged 50 years or older (58.5%), whereas 24.4% were aged 19–49 years and 17.1% were aged 18 years or younger. Across the seven disability-type categories, internal/facial disability (22.4%) and hearing/speech disability (18.6%) were the most common, followed by brain lesion disability (16.0%), physical disability (15.9%), visual disability (12.4%), intellectual/autistic disability (9.3%), and psychiatric disability (5.4%). Just over half of participants (53.0%) were classified as having a severe disability under the Korean Disability Registration System. Educational attainment was concentrated at the high-school level (35.9%), followed by middle school (16.8%), elementary school (14.5%), and college/university education or higher (15.0%). A total of 85.4% had not undergone disability re-evaluation before Wave 1, whereas the remaining 14.6% reported re-evaluation experiences primarily due to periodic reassessment (10.1%) or other administrative reasons. Detailed baseline characteristics are presented in Table 1.

### 3.2. Response Retention and Descriptive Statistics

Of the 6121 panel members enrolled in Wave 1, 5527 (90.3%) responded in Wave 2, with response rates declining gradually to 4702 (76.8%) in Wave 7. A total of 4283 participants (70.0%) responded to all seven waves. Item-level non-response for the disability acceptance and unmet healthcare needs variables was minimal, with response rates closely matching the overall wave-level retention rates across all waves.

Descriptive statistics for the focal variables across the seven waves are presented in Table 2. Mean disability acceptance remained relatively stable from Wave 1 through Wave 5 (range: 2.305–2.309 on a 1–4 scale), then increased at Wave 6 (M = 2.419) and Wave 7 (M = 2.454). Internal consistency of the 12-item disability acceptance scale was consistently good across all seven waves (Cronbach’s α range: 0.808–0.823). The prevalence of unmet healthcare needs declined gradually from 4.4% at Wave 1 to approximately 2.0% from Wave 5 onward, consistent with previously reported national trends during the same period [2].

Within-wave correlations between disability acceptance and unmet healthcare needs were consistently negative and statistically significant across all seven waves (r range: −0.036 to −0.088, all *p* < 0.05), indicating that participants with higher disability acceptance tended to report lower levels of unmet healthcare needs at each measurement occasion. Although these correlations were small in magnitude, the consistency of the association across all seven waves supported the stability of the observed relationship between the two constructs.

Inter-wave correlations among disability acceptance scores indicated high temporal stability from Wave 1 through Wave 5 (adjacent-wave r range: 0.956–0.976), followed by a marked decrease at the Wave 5-to-Wave 6 transition (*r* = 0.497), with partial recovery at the Wave 6-to-Wave 7 transition (*r* = 0.656). Importantly, internal consistency reliability remained stable at Waves 6 and 7 (α = 0.808 and 0.809, respectively), suggesting that the reduction in inter-wave association was unlikely to reflect deterioration of the measurement instrument. Potential explanations for this pattern are discussed later.

### 3.3. Random Intercept Cross-Lagged Panel Model

#### 3.3.1. Model Fit

The freely estimated RI-CLPM (with autoregressive and cross-lagged coefficients allowed to vary across the six wave-to-wave transitions) fit the data well: χ^2^(57) = 299.26, *p* < 0.001; CFI = 0.996; TLI = 0.994; RMSEA = 0.026, 90% CI [0.023, 0.029]; and SRMR = 0.020. All fit indices exceeded conventional criteria for good fit [14]. As reported in Section 2.3.2, a likelihood-ratio test indicated that the freely estimated model fit significantly better than the time-invariant specification, scaled Δχ^2^(20) = 838.25, *p* < 0.001.

#### 3.3.2. Between-Person Associations (Random Intercept Correlation)

The estimated covariance between the disability acceptance random intercept and the unmet healthcare needs random intercept was statistically significant and negative (estimated covariance = −0.002, SE = 0.001, *p* = 0.001), corresponding to a standardized correlation of r = −0.132. This finding indicates that, after accounting for within-person variation, participants with higher trait-level disability acceptance tended to report lower overall levels of unmet healthcare needs across the seven-year observation period. Although small in magnitude, this between-person association was consistent with the within-wave correlations reported in Section 3.2 and suggested an inverse association between the two constructs at the trait level.

#### 3.3.3. Within-Person Autoregressive Stability

Standardized autoregressive coefficients for disability acceptance were large and statistically significant across the five Wave 1–Wave 5 transitions (standardized βs ranging from 0.935 to 0.963), indicating high temporal stability during the first five years of observation. The autoregressive coefficient decreased markedly at the Wave 5-to-Wave 6 transition (*β* = 0.212, *p* < 0.001) and partially recovered at the Wave 6-to-Wave 7 transition (*β* = 0.439, *p* < 0.001). For unmet healthcare needs, autoregressive coefficients were small in magnitude but statistically significant at the Wave 1-to-Wave 2 (*β* = 0.115, *p* < 0.001), Wave 2-to-Wave 3 (*β* = 0.106, *p* = 0.002), and Wave 3-to-Wave 4 (*β* = 0.100, *p* = 0.012) transitions, becoming non-significant thereafter (Wave 4-to-Wave 5: *β* = 0.045, *p* = 0.256; Wave 5-to-Wave 6: *β* = 0.047, *p* = 0.281; Wave 6-to-Wave 7: *β* = 0.013, *p* = 0.784).

#### 3.3.4. Within-Person Cross-Lagged Associations

**Figure 2:**
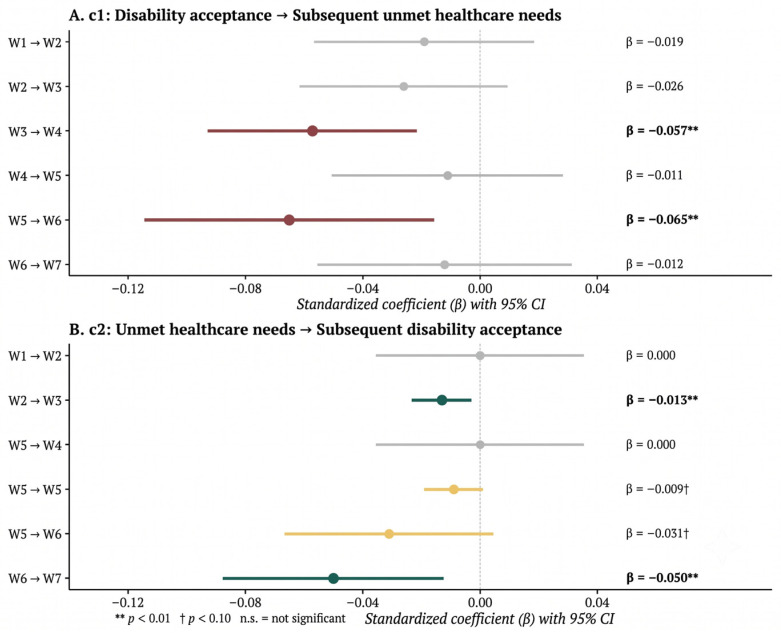
Standardized Cross-Lagged Associations Between Disability Acceptance and Unmet Healthcare Needs Across the Six Wave-to-Wave Transitions. Panel (**A**) displays paths from disability acceptance to subsequent unmet healthcare needs (c1), whereas Panel (**B**) displays reciprocal paths from unmet healthcare needs to subsequent disability acceptance (c2). The vertical dashed line represents the null effect (*β* = 0). Red and green markers indicate statistically significant associations (** *p* < 0.01), yellow markers indicate marginally significant associations († *p* < 0.10), and gray markers indicate non-significant associations.

Cross-lagged coefficients quantifying the prospective within-person associations between disability acceptance and unmet healthcare needs are presented in Table 3 and visualized in Figure 2. The path from disability acceptance to subsequent unmet healthcare needs (c1) was negative across all six transitions but statistically significant at two transitions: Wave 3-to-Wave 4 (*β* = −0.057, *p* = 0.002) and Wave 5-to-Wave 6 (*β* = −0.065, *p* = 0.008). The reciprocal path from unmet healthcare needs to subsequent disability acceptance (c2) was also negative across most transitions, with statistically significant associations at the Wave 2-to-Wave 3 transition (*β* = −0.013, *p* = 0.008) and the Wave 6-to-Wave 7 transition (*β* = −0.050, *p* = 0.008), and marginal significance at the Wave 4-to-Wave 5 (*p* = 0.054) and Wave 5-to-Wave 6 (*p* = 0.080) transitions. Figure 2 displays the standardized cross-lagged estimates and their corresponding 95% confidence intervals across the six wave-to-wave transitions.

Taken together, these findings suggest that within-person associations between disability acceptance and unmet healthcare needs were not constant across time but emerged selectively during particular wave-to-wave transitions. Although the individual cross-lagged coefficients were modest in magnitude, the consistently negative direction of the coefficients across transitions indicated a stable pattern of inverse association between the two constructs at the within-person level.

#### 3.3.5. Final Pathway Model

**Figure 3:**
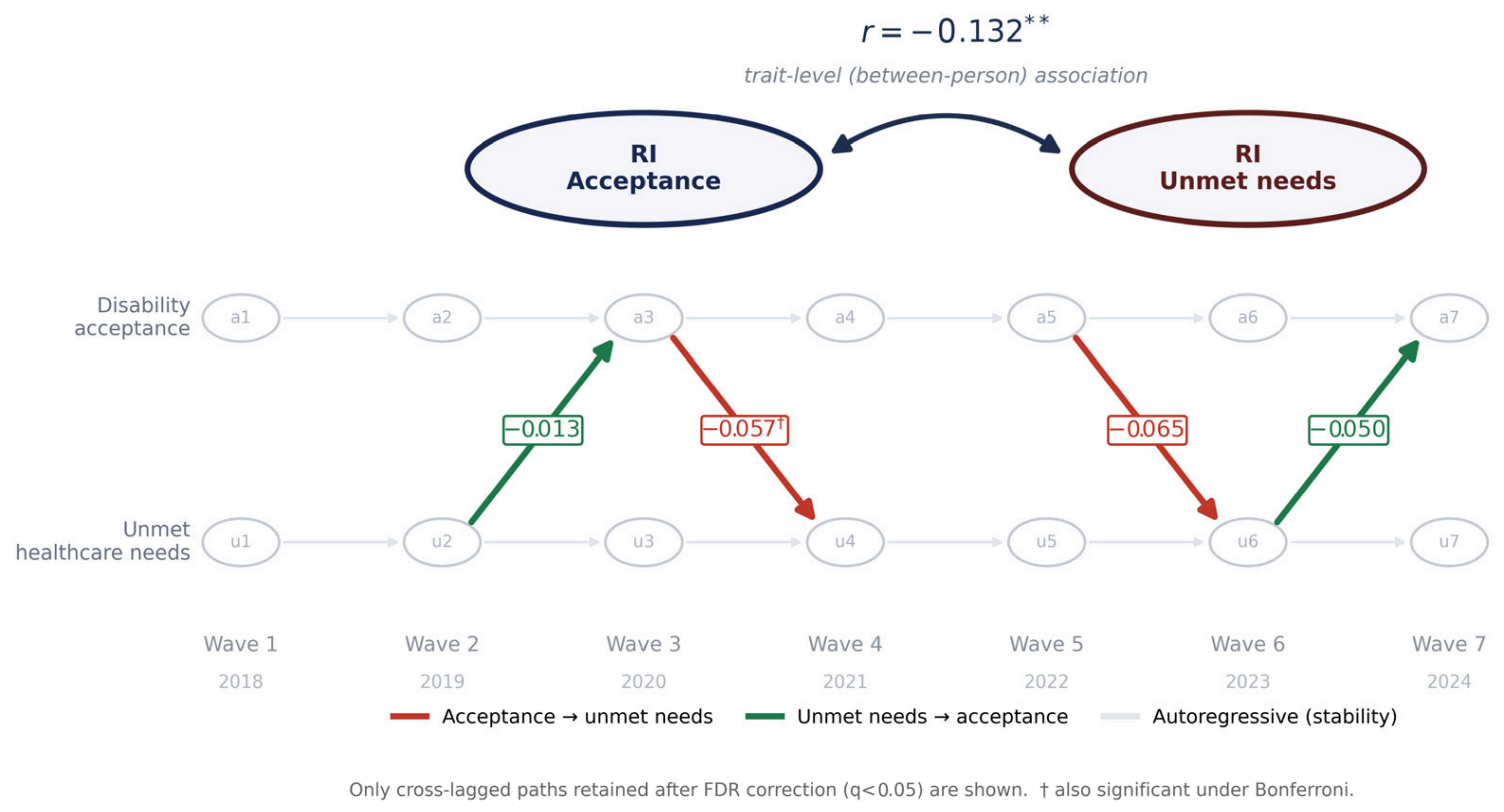
Final RI-CLPM showing only statistically retained pathways after FDR correction. Standardized coefficients are shown beside each path; r = −0.132 denotes the between-person random-intercept correlation (** *p* < 0.01, † *p* < 0.10).

Figure 3 summarizes the statistically retained RI-CLPM. At the between-person level, disability acceptance and unmet healthcare needs showed a stable inverse association (r = −0.132). At the within-person level, four cross-lagged paths remained significant after FDR correction; all retained effects were negative, indicating that higher acceptance predicted lower subsequent unmet healthcare needs, and vice versa.

### 3.4. Supplementary and Robustness Analyses

McNemar tests comparing adjacent waves indicated that the decline in unmet healthcare needs was concentrated in the early observation period (Wave 1 to 2: χ^2^(1) = 6.17, *p* = 0.013; Wave 2 to 3: χ^2^(1) = 15.08, *p* < 0.001), with no significant adjacent-wave changes from Wave 3 onward (all *p* ≥ 0.080) and a significant cumulative decline from Wave 1 to Wave 7 (χ^2^(1) = 42.74, *p* < 0.001). Design-weighted prevalence estimates declined from 5.13% (95% CI [4.12, 6.14]) at Wave 1 to 1.94% (95% CI [1.35, 2.53]) at Wave 7, mirroring the unweighted trajectory.

After Benjamini–Hochberg FDR correction across the twelve cross-lagged tests, four paths remained significant (*q* < 0.05): the acceptance-to-unmet-needs paths at Waves 3 to 4 and 5 to 6, and the unmet-needs-to-acceptance paths at Waves 2 to 3 and 6 to 7. Under the more conservative Bonferroni criterion, the Wave 3-to-4 acceptance-to-unmet-needs path remained significant (corrected *p* = 0.023).

The RI-CLPM was preferred over the traditional cross-lagged panel model on both information criteria (AIC: −49,681.6 vs. −49,111.6; BIC: −49,265.0 vs. −48,715.1) and on global fit (CFI = 0.996 vs. 0.987; RMSEA = 0.026 vs. 0.052). In the categorical WLSMV sensitivity model (*n* = 4283), the two acceptance-to-unmet-needs transitions identified in the primary model remained negative (Wave 3 to 4: b = −0.35, *p* = 0.027; Wave 5 to 6: b = −0.66, *p* < 0.001, the latter surviving FDR correction), supporting the robustness of the direction and locus of the within-person associations under a categorical treatment of the outcome.

Targeted longitudinal measurement invariance testing across Waves 5 and 6 (dual-wave respondents, *n* = 4706) supported metric and scalar invariance by the ΔCFI ≤ 0.01 criterion (metric: ΔCFI = −0.002; scalar: ΔCFI = −0.004), although scaled likelihood-ratio tests were statistically significant at this sample size. Score tests localized intercept non-invariance primarily to the reverse-worded items (Items 12, 7, and 3, together with Item 11), and a partial-scalar model freeing these four intercepts continued to indicate a significant latent mean increase at Wave 6 (Δ = 0.105, *p* < 0.001). These results indicate that the Wave 5–6 discontinuity reflects item-intercept drift concentrated among negatively worded items rather than a change in the construct’s meaning (see Section 4.3).

Wave 7 non-response was unrelated to baseline unmet healthcare needs (adjusted OR = 1.07, 95% CI [0.81, 1.43], *p* = 0.637; unadjusted OR = 1.13, *p* = 0.383) but was significantly associated with lower baseline disability acceptance, male sex, and severe disability. Missingness was therefore related to observed baseline covariates rather than to the outcome itself, a pattern consistent with the missing-at-random assumption underlying FIML estimation.

When baseline sex, disability severity, age group, and educational attainment were included as predictors of the random intercepts, the within-person cross-lagged pattern was preserved (the Wave 3-to-4 and 5-to-6 acceptance-to-unmet-needs paths and the Wave 2-to-3 unmet-needs-to-acceptance path remained significant after FDR correction). In contrast, the residual random-intercept correlation was attenuated and non-significant (*r* = −0.039, *p* = 0.770), indicating that sociodemographic and disability-related characteristics substantially account for the trait-level association. Wald tests indicated that the average acceptance-to-unmet-needs coefficient did not differ between pandemic-era (2019–2022) and non-pandemic transitions in the covariate-adjusted model (χ^2^(1) = 0.00, *p* = 0.975). The reverse direction was sensitive to the treatment of the two transitions affected by the Wave 6–7 intercept drift (*p* = 0.023 when these transitions were included; *p* = 0.846 when the comparison was restricted to drift-free transitions), and pandemic-related interpretations of that direction are therefore offered with caution.

## 4. Discussion

This study examined the bidirectional longitudinal associations between disability acceptance and unmet healthcare needs among newly registered persons with disabilities in South Korea using seven annual waves of nationally representative panel data (2018–2024). To our knowledge, this is the first study to apply the RI-CLPM to the DLDP, enabling the explicit separation of stable between-person differences from within-person dynamics over a seven-year observation window encompassing the pre-pandemic, pandemic, and post-pandemic periods. Three findings warrant detailed discussion.

### 4.1. Trait-Level Inverse Association Between Disability Acceptance and Unmet Healthcare Needs

At the between-person (trait) level, the random intercept correlation between disability acceptance and unmet healthcare needs was statistically significant and negative (r = −0.132, *p* = 0.001). This finding indicates that, after partialling out within-person fluctuations and measurement-occasion-specific factors, individuals with stable higher levels of disability acceptance over the seven years also tended to be those with stable lower levels of unmet healthcare needs. The direction of this association mirrors the seven within-wave correlations reported in Section 3.2, all of which were significantly negative.

This pattern is consistent with the broader literature on psychosocial adaptation to chronic illness and disability [6]. It aligns with the Andersen Behavioral Model framework [19], which posits that individual predisposing factors (including psychological resources such as disability acceptance) shape healthcare utilization behavior alongside enabling and need factors. Within the Korean context, prior research using DLDP data has documented persistent unmet healthcare needs among persons with disabilities [1,2], but has not previously demonstrated the role of disability acceptance as a stable trait-level correlate of these needs. Our finding extends Kim and colleagues’ [7] earlier demonstration that activities-of-daily-living limitations predict unmet healthcare needs by showing that psychological adaptation to disability, independent of functional limitations, is also linked to trait-level disparities in healthcare access.

Although the absolute magnitude of the trait-level correlation was modest, its statistical robustness in a sample of 6121 panel members and the consistent direction across all seven measurement occasions suggest that disability acceptance and unmet healthcare needs are consistently associated at the between-person level over the observation period. Supplementary covariate-adjusted models, however, indicated that this trait-level association was substantially explained by sociodemographic and disability-related characteristics, particularly age, disability severity, and educational attainment (Section 3.4); it should therefore be read as a descriptive between-person association rather than an independently adjusted relation. This trait-level finding may have implications for the design of population health interventions, which we address in Section 4.5.

### 4.2. Bidirectional Within-Person Effects Emerge Selectively Across Time

Cross-lagged within-person associations were small in magnitude but statistically significant for four of the twelve estimated transitions, with coefficients consistently negative. The path from disability acceptance to subsequent unmet healthcare needs (c1) was significant at the Wave 3-to-Wave 4 transition (*β* = −0.057) and the Wave 5-to-Wave 6 transition (*β* = −0.065), while the reverse path from unmet healthcare needs to subsequent disability acceptance (c2) was significant at the Wave 2-to-Wave 3 transition (*β* = −0.013) and the Wave 6-to-Wave 7 transition (*β* = −0.050). Two additional c2 transitions (Wave 4-to-Wave 5 and Wave 5-to-Wave 6) approached statistical significance (0.05 < *p* < 0.10).

These findings suggest that disability acceptance and unmet healthcare needs were reciprocally associated at the within-person level, although the associations were not constant across time. Rather than operating as a continuous process throughout the seven-year observation window, the cross-lagged associations emerged most clearly at transitions coinciding with broader changes in the healthcare environment in South Korea, including the onset and intensification of the COVID-19 pandemic (Wave 3-to-Wave 4: 2020 to 2021) and the post-pandemic recovery period (Wave 6-to-Wave 7: 2023 to 2024). This temporal patterning is consistent with Akobirshoev and colleagues’ [20] finding that the pandemic disproportionately increased unmet healthcare needs among adults with disabilities in the United States and may indicate that periods of healthcare system disruption strengthen associations that are less detectable during periods of relative stability.

A second interpretive consideration is that the within-person associations, although statistically significant, were of modest magnitude in standardized terms (β = −0.013 to −0.065). This pattern is consistent with prior RI-CLPM applications in adjacent domains, which have repeatedly reported that within-person cross-lagged estimates are typically smaller than conventional cross-lagged estimates because the RI-CLPM removes stable between-person variance that may inflate traditional cross-lagged associations [8,9]. Even modest within-person associations may have meaningful implications when accumulated across large populations and extended observation periods.

The practical significance of these coefficients should be evaluated against empirical benchmarks for cross-lagged parameters rather than against conventional standards for bivariate correlations. Orth and colleagues [21], drawing on the distribution of published cross-lagged estimates, proposed standardized values of approximately 0.03, 0.07, and 0.12 as small, medium, and large benchmarks, respectively; by these standards, the significant paths observed here (|β| = 0.013–0.065) range from small to approaching medium. Adachi and Willoughby [22] further demonstrated that cross-lagged coefficients estimated while controlling for prior levels of the outcome are necessarily compressed, particularly when autoregressive stability is high, as it was for disability acceptance in the present data, so that small standardized values in this context can correspond to non-trivial cumulative change. From a population-health perspective, small associations can be consequential when they operate repeatedly over time or across large populations [23]; both conditions apply here, as the associations recurred across a seven-year window and Korea’s registered disability population exceeds 2.6 million persons. If the observed within-person association reflects a modifiable process, an association of this magnitude would correspond to a meaningful number of persons whose access to needed care co-varies with psychosocial adaptation. At the same time, because these are observational longitudinal associations, such projections indicate the potential scope for intervention rather than an expected causal yield.

### 4.3. Stability of Disability Acceptance and the Wave 5–6 Discontinuity

Autoregressive coefficients for disability acceptance were very large from Wave 1 through Wave 5 (standardized βs ranging from 0.935 to 0.963), indicating that within-person variation in disability acceptance was highly persistent during the first five years following panel enrollment. Such strong temporal stability is consistent with conceptualizing disability acceptance as a relatively enduring psychosocial adaptation construct rather than a highly state-dependent variable [5].

Importantly, high autoregressive stability in the RI-CLPM framework does not preclude the interpretation of cross-lagged associations. Rather, statistically significant cross-lagged associations observed under conditions of strong autoregressive stability may indicate that within-person deviations show detectable prospective associations despite substantial temporal continuity. In this respect, the observed cross-lagged coefficients may represent relatively conservative estimates of within-person longitudinal associations. Importantly, the presence of statistically significant cross-lagged associations despite strong autoregressive stability suggests that the RI-CLPM retained meaningful within-person variability beyond simple autoregressive persistence.

At the Wave 5-to-Wave 6 transition, however, the autoregressive coefficient decreased markedly (β = 0.212), with partial recovery at the Wave 6-to-Wave 7 transition (β = 0.439). This discontinuity warrants careful consideration. Several lines of evidence suggest that the observed reduction is unlikely to be attributable to deterioration of the measurement instrument: (a) the 12-item disability acceptance scale retained good internal consistency at Waves 6 and 7 (Cronbach’s α = 0.808 and 0.809, respectively), comparable to that observed at Waves 1 through 5 (α range: 0.813 to 0.823); (b) the value range, response option labels, and item wording of the disability acceptance items were identical across all seven waves of the DLDP; and (c) the inter-wave correlation pattern showed an abrupt rather than gradual decline, which would be less consistent with gradual measurement drift.

Consistent with this interpretation, the targeted invariance analyses reported in Section 3.4 localized the discontinuity to item-intercept drift concentrated in the reverse-worded items, with factor loadings invariant across Waves 5 and 6 and the latent mean increase persisting under a partial-scalar specification. Any residual discontinuity beyond this measurement-level evidence may reflect unobserved administrative factors that cannot be verified with the available documentation, and future DLDP-based research may benefit from obtaining records of year-specific operational procedures from KODDI. Despite this uncertainty, the discontinuity does not necessarily invalidate the cross-lagged interpretations reported above, because the freely estimated RI-CLPM specification accommodates wave-specific differences in stability without imposing uniform autoregressive constraints.

### 4.4. Limitations

Several limitations should be considered when interpreting the present findings. First, the DLDP classifies disability type and severity based on the panel enrollment period (2015–2017) and treats these variables as time-invariant across subsequent waves. As a result, later changes related to disability re-evaluation or changes in functional status were not reflected in the analytic models. Future longitudinal disability panels may benefit from incorporating time-varying indicators of disability status.

Second, unmet healthcare needs were assessed using a single dichotomous item. Although this approach is consistent with prior DLDP-based studies [2,7] and national health survey practice in Korea, it did not distinguish among specific causes of unmet healthcare needs (e.g., financial, geographic, transportation-related, or provider-related barriers). In addition, the relatively low prevalence of unmet healthcare needs across waves may have reduced sensitivity for detecting smaller within-person associations.

Third, the DLDP cohort consists of individuals who had newly acquired disability registration between 2015 and 2017. Accordingly, the findings may not fully generalize to persons with long-term disability registration histories or to healthcare systems outside the Korean context.

Fourth, although the RI-CLPM accounts for stable between-person differences, the analyses did not include time-varying covariates that may influence both disability acceptance and unmet healthcare needs over time, such as changes in socioeconomic conditions, caregiving arrangements, or pandemic-related experiences. Future studies incorporating time-varying contextual factors may help clarify the longitudinal associations observed in the present study.

Fifth, although the DLDP employs a stratified two-stage complex sampling design, the primary RI-CLPM was estimated without sampling weights, consistent with model-based longitudinal SEM practice. Design-weighted prevalence estimates (Section 3.4) showed the same declining trajectory as the unweighted estimates. Still, unweighted model parameters may not fully generalize to the national population of newly registered persons with disabilities, and this should be considered when interpreting the findings.

Sixth, a marked reduction in the autoregressive stability coefficient for disability acceptance was observed between Waves 5 and 6. Although the freely estimated RI-CLPM specification accommodated this variation and supplementary analyses yielded substantively comparable patterns, the possibility of unmeasured administrative or contextual influences during this transition cannot be entirely ruled out.

Finally, relying on a fixed one-year time lag may either miss short-term dynamics or long-term cumulative effects between disability acceptance and healthcare access. Future studies should utilize varying time intervals or continuous-time models to explore optimal temporal windows.

### 4.5. Implications for Policy and Practice

The present findings have three implications for healthcare policy and disability service practice in Korea. First, the trait-level association between disability acceptance and unmet healthcare needs suggests that healthcare access interventions for persons with disabilities may benefit from incorporating components that address psychosocial adaptation alongside structural barriers, such as financial assistance and physical accessibility. Routine disability services in Korea, including those administered through the Korea Disabled People’s Development Institute and local disability welfare centers, may consider incorporating brief disability acceptance assessments at intake and offering linkage to counseling, peer support, or adaptive skills programming for individuals with lower levels of disability acceptance.

Second, the selective emergence of within-person cross-lagged associations during the COVID-19 and post-pandemic transitions underscores the importance of disability-responsive healthcare system planning during periods of systemic stress. The pandemic experience highlighted that persons with disabilities face heightened risks of healthcare access disruption when general healthcare delivery is destabilized [20]. Healthcare systems may benefit from developing contingency protocols to maintain continuity of care for persons with disabilities during future public health emergencies, and the Korean Ministry of Health and Welfare’s Disability and Health Policy Division may consider periodic reviews of metrics for the provision of accessible care.

Third, the gradual reduction in unmet healthcare needs from 4.4% at Wave 1 to 2.0% at Wave 7 is a notable population-level finding that converges with national-level health statistics. However, our analysis suggests that even among individuals experiencing reduced unmet healthcare needs at the population level, within-person associations between disability acceptance and healthcare access remain selectively observed over time. Sustained policy attention to the psychosocial dimensions of disability adaptation, rather than exclusive focus on structural healthcare access metrics, may help support the long-term well-being of persons with disabilities across their life course.

## 5. Conclusions

In a seven-year nationally representative panel of newly registered persons with disabilities in South Korea, disability acceptance and unmet healthcare needs were inversely associated at the trait (between-person) level, and small but reciprocal within-person cross-lagged associations emerged selectively during transitions that coincided with the COVID-19 pandemic and its aftermath. The findings suggest potential value in integrating psychosocial adaptation components into healthcare access interventions for persons with disabilities, and they highlight the importance of disability-responsive contingency planning during periods of healthcare system disruption. Future research should extend the RI-CLPM framework to incorporate time-varying confounders, examine differential associations by disability type and severity, and link DLDP data with administrative healthcare utilization records to clarify the mechanisms underlying the reciprocal associations observed in the present study. These findings suggest that interventions aimed at reducing unmet healthcare needs among persons with disabilities may be strengthened by integrating psychosocial adaptation support with conventional healthcare access strategies.

## Figures and Tables

**Table 1:** Baseline (Wave 1) sample characteristics (N = 6121).

Characteristic		*n*	%
Sex	Male	3389	55.4
	Female	2732	44.6
Age category	≤18 years	1047	17.1
	19–49 years	1493	24.4
	≥50 years	3581	58.5
Disability type	Physical disability	974	15.9
	Brain lesion disability	980	16.0
	Visual disability	757	12.4
	Hearing/speech disability	1141	18.6
	Intellectual/autism disability	567	9.3
	Mental disability	328	5.4
	Internal/facial disability	1374	22.4
Disability severity	Severe	3242	53.0
	Mild	2879	47.0
Educational attainment	Pre-school	550	9.0
	Elementary school	888	14.5
	Middle school	1027	16.8
	High school	2197	35.9
	Junior college	282	4.6
	Bachelor’s degree	570	9.3
	Master’s degree	57	0.9
	Doctoral degree	14	0.2
	No formal education	536	8.8
Prior disability re-evaluation	None	5225	85.4
	Periodic reassessment	621	10.1
	Other reasons	275	4.5

Note. Sex and educational attainment refer to self-reported status at Wave 1 (2018). Disability type, severity, and age category were based on the sampling-design classification at panel enrollment (2015–2017). “Other reasons” for prior re-evaluation combine registration error (1.3%), disability pension application (1.0%), acquisition of an additional disability (0.9%), application for personal assistance services (0.4%), and miscellaneous/unspecified responses (1.0%).

**Table 2:** Descriptive statistics and within-wave correlations of focal variables by wave.

Variable	Wave	N	M (SD)	Min	Max	*α*	*r* (DA × UHN)	*p*
Disability acceptance	1	6121	2.31 (0.45)	1.00	4.00	0.813	—	—
	2	5527	2.31 (0.45)	1.00	4.00	0.814	—	—
	3	5259	2.31 (0.45)	1.00	4.00	0.816	—	—
	4	5024	2.31 (0.46)	1.00	4.00	0.823	—	—
	5	4904	2.31 (0.45)	1.00	4.00	0.822	—	—
	6	4796	2.42 (0.43)	1.00	3.92	0.808	—	—
	7	4702	2.45 (0.42)	1.00	4.00	0.809	—	—
Unmet healthcare needs	1	6121	0.04 (0.21)	0	1	—	−0.068	<0.001
	2	5527	0.04 (0.19)	0	1	—	−0.050	<0.001
	3	5259	0.03 (0.16)	0	1	—	−0.059	<0.001
	4	5024	0.03 (0.16)	0	1	—	−0.082	<0.001
	5	4904	0.02 (0.14)	0	1	—	−0.039	0.007
	6	4796	0.02 (0.14)	0	1	—	−0.088	<0.001
	7	4702	0.02 (0.14)	0	1	—	−0.036	0.013

Note. Disability acceptance is the mean of 12 items scored on a 4-point Likert scale (1–4); higher scores indicate greater acceptance. Unmet healthcare needs were assessed using a single dichotomous item (1 = experienced; 0 = did not experience). *α* = Cronbach’s alpha for disability acceptance; r (Disability Acceptance × Unmet Healthcare Needs) = within-wave Pearson correlation between disability acceptance and unmet healthcare needs. Pearson point-biserial correlations were computed because unmet healthcare needs were coded as a dichotomous variable. Mean values for unmet healthcare needs correspond to the proportion answering “yes” (Waves 1–7: 4.4%, 3.7%, 2.6%, 2.5%, 2.0%, 1.9%, and 2.0%, respectively); McNemar tests of adjacent-wave changes and design-weighted prevalence estimates are reported in Section 3.4.

**Table 3:** Standardized autoregressive and cross-lagged coefficients from the Random Intercept Cross-Lagged Panel Model.

Variable	Transition	*b*	SE	*z*	*p*	*β*	95% CI (*β*)
Autoregressive: Acceptance	W1 → W2	0.938	0.008	114.87	<0.001	0.935	[0.923, 0.948]
	W2 → W3	0.966	0.005	180.65	<0.001	0.963	[0.954, 0.971]
	W3 → W4	0.956	0.007	136.29	<0.001	0.955	[0.945, 0.964]
	W4 → W5	0.951	0.006	151.09	<0.001	0.963	[0.956, 0.969]
	W5 → W6	0.198	0.036	5.45	<0.001	0.212	[0.138, 0.286]
	W6 → W7	0.410	0.031	13.25	<0.001	0.439	[0.377, 0.501]
Autoregressive: Unmet needs	W1 → W2	0.104	0.026	4.05	<0.001	0.115	[0.061, 0.169]
	W2 → W3	0.087	0.028	3.11	0.002	0.106	[0.041, 0.172]
	W3 → W4	0.097	0.039	2.51	0.012	0.100	[0.023, 0.177]
	W4 → W5	0.040	0.035	1.14	0.256	0.045	[−0.032, 0.122]
	W5 → W6	0.045	0.042	1.08	0.281	0.047	[−0.038, 0.132]
	W6 → W7	0.013	0.048	0.27	0.784	0.013	[−0.077, 0.103]
Cross-lagged: Acceptance → Unmet needs (c1)	W1 → W2	−0.009	0.009	−1.03	0.302	−0.019	[−0.055, 0.017]
	W2 → W3	−0.011	0.008	−1.40	0.162	−0.026	[−0.062, 0.010]
	W3 → W4	−0.022	0.007	−3.10	0.002	−0.057	[−0.091, −0.022]
	W4 → W5	−0.004	0.007	−0.55	0.585	−0.011	[−0.050, 0.028]
	W5 → W6	−0.022	0.009	−2.64	0.008	−0.065	[−0.112, −0.019]
	W6 → W7	−0.005	0.008	−0.54	0.587	−0.012	[−0.054, 0.031]
Cross-lagged: Unmet needs → Acceptance (c2)	W1 → W2	0.000	0.009	−0.02	0.984	0.000	[−0.010, 0.010]
	W2 → W3	−0.027	0.010	−2.64	0.008	−0.013	[−0.023, −0.003]
	W3 → W4	0.000	0.010	0.01	0.989	0.000	[−0.008, 0.008]
	W4 → W5	−0.023	0.012	−1.92	0.054	−0.009	[−0.019, 0.000]
	W5 → W6	−0.081	0.046	−1.75	0.080	−0.031	[−0.066, 0.004]
	W6 → W7	−0.127	0.048	−2.65	0.008	−0.050	[−0.088, −0.012]
Random intercept correlation	RI_Acceptance ↔ RI_Unmet needs	−0.002	0.001	—	0.001	−0.132	[−0.211, −0.053]

Note. N = 6121. Estimation used robust maximum likelihood (MLR) with full-information maximum likelihood (FIML) for missing data. *b* = unstandardized coefficient; *β* = standardized estimate. Model fit: χ^2^(57) = 299.26, *p* < 0.001; Comparative Fit Index (CFI) = 0.996; Tucker–Lewis Index (TLI) = 0.994; Root Mean Square Error of Ap-proximation (RMSEA) = 0.026, 90% confidence interval (CI) [0.023, 0.029]; Stand-ardized Root Mean Square Residual (SRMR) = 0.020. 95% CI (*β*) = 95% confidence interval for the standardized estimate, obtained from the standardized solution via the delta method. Explained variance (R^2^) for the within-person components: disability acceptance = 0.875–0.928 (Waves 2–5), 0.046 (Wave 6), and 0.199 (Wave 7); unmet healthcare needs = 0.000–0.014 (Waves 2–7).

## Data Availability

The Disability and Life Dynamics Panel (DLDP) data are available from the Korea Disabled People’s Development Institute (KODDI) through the Disability Statistics Data Portal upon reasonable request and completion of the registered data access procedure (https://koddi.or.kr/stat/html/user/pwpn/daap/app/pwdlPnlAplyPrcd.do, accessed on 31 July 2026).

## Abbreviations

The following abbreviations are used in this manuscript:

RI-CLPM	Random Intercept Cross-Lagged Panel Model
CLPM	Cross-Lagged Panel Model
DLDP	Disability and Life Dynamics Panel
KODDI	Korea Disabled People’s Development Institute
FIML	Full-Information Maximum Likelihood
MLR	Robust Maximum Likelihood
WLSMV	Weighted Least Squares with Mean and Variance Adjustment
FDR	False Discovery Rate
MAR	Missing at Random
CFI	Comparative Fit Index
TLI	Tucker–Lewis Index
RMSEA	Root Mean Square Error of Approximation
SRMR	Standardized Root Mean Square Residual
